# Evidence of colistin resistance genes (*mcr-1* and *mcr-2*) in wild birds and its public health implication in Egypt

**DOI:** 10.1186/s13756-019-0657-5

**Published:** 2019-12-03

**Authors:** Zeinab S. Ahmed, Esraa A. Elshafiee, Hanan S. Khalefa, Mona Kadry, Dalia A. Hamza

**Affiliations:** 1 Department of Zoonoses, Faculty of Veterinary Medicine, Cairo University, Box 12211, Giza, PO Egypt; 2 Department of Veterinary Hygiene and Management, Faculty of Veterinary Medicine, Cairo University, Box 12211, Giza, PO Egypt

**Keywords:** Wild bird, *Enterobacteriaceae*, *P. aeruginosa*, *mcr* genes, Egypt

## Abstract

**Background:**

Antimicrobial resistance has become one of the most severe global threats to human and veterinary Medicin*e. coli*stin is an effective therapeutic agent against multi-drug-resistant pathogens. However, the discovery of transferable plasmids that confer resistance to colistin (*mcr-1)* has led to challenges in medical science. This study describes the role of wild birds in the harbouring and environmental spread of colistin-resistant bacteria, which could pose a potential hazard to human and animal health.

**Methods:**

In total, 140 faecal samples from wild birds (migratory and resident birds) were tested. Twenty surface water samples were collected from the area in which wild bird trapping was conducted, and 50 human stool samples were collected from individuals residing near the surface water sources and farm buildings. Isolation and identification of *Enterobacteriaceae* and *Pseudomonas aeruginosa* from the different samples were performed using conventional culture techniques and biochemical identification. PCR amplification of the *mcr* genes was performed in all positive isolates. Sequencing of *mcr*-1 genes from three randomly selected *E. coli* carrying mcr-1 isolates; wild birds, water and humans was performed.

**Result:**

The bacteriological examination of the samples showing isolates of *Escherichia coli*, *Klebsiella pneumoniae*, *Klebsiella oxytoca* and *P. aeruginosa*. The results of multiplex PCR of the *mcr* genes revealed that *E. coli* was the most prevalent gram-negative bacterium harbouring the *mcr* genes, whereas a low prevalence was observed for *K. pneumoniae*. The prevalence of *mcr-*1 in resident birds, migratory birds, water sources and humans were 10.4, 20,16.6 and 9.6% while the prevalence of *mcr*-2 were 1.4, 3.6, 11.1 and 9.6%, respectively. Sequencing of the *mcr-1* gene from the three *E. coli* carrying *mcr*-*1* isolates indicated a possible correlation between the wild bird and surface water isolates.

**Conclusion:**

The detection of *mcr*-1-positive bacteria in wild birds in Egypt indicates the possible environmental dissemination of this gene through bird activity. The impact of the interaction between domestic and wild animals on public health cannot be overlooked.

## Background

Antimicrobial resistance (AMR) has become one of the most severe global threats to human and veterinary medicine. This crisis has been catalysed by the rapid emergence of carbapenemase-producing *Enterobacteriaceae* expressing enzymes such as KPC-2 (*Klebsiella pneumoniae* carbapenemase-2) and NDM-1 (New Delhi metallo-β-lactamase-1) [1, 2].

Colistin is considered the last resort for the treatment of severe infections caused by multi-drug-resistant bacteria. Thus, the global increase in carbapenemase-producing *Enterobacteriaceae* has resulted in the overuse of colistin with the inevitable risk of emerging resistance [3].

Recently, resistance to colistin has been linked to not only chromosomal mutations but also plasmid-mediated mechanisms [4, 5]. Unfortunately, the presence of colistin resistance on mobile genetic elements poses a significant public health risk, as these elements can spread rapidly via horizontal transfer [6]. A transferable plasmid that confers resistance to colistin (*movable colistin resistance 1*, *mcr-1*) was first discovered in *Escherichia coli, K. pneumoniae* and *Pseudomonas aeruginosa* strains from China between April 2011 and November 2014 [7]. In addition, the *mcr-1* gene has also been observed on plasmids containing other AMR genes, such as genes encoding carbapenemases [8, 9] and extended-spectrum β-lactamases [10–12]. Many studies have attributed the emergence and silent dissemination of plasmid genes involved in polymyxin resistance to the current use of colistin as an antibiotic growth promoter (AGP) in livestock [13].

The presence of bacteria of potential zoonotic importance among migratory and non-migratory wild birds has public health significance. The bird migration provides a mechanism for the establishment of new endemic foci of disease at great distances from the site of acquisition of and infection [14]. Therefore, migratory and non-migratory wild birds could serve as reservoirs of resistant bacteria and genetic factors associated with AMR [15].

Although the origin of bacterial resistance genes in wild animals remains unclear, as wildlife are not exposed to antibiotics directly, contact with sewage or animal manure might a possible source [16]. Moreover, contact with water and feeding habits seem to be the main factors affecting the transmission of resistant bacteria from humans or veterinary sources to wild animals [17, 18]. This gap in knowledge highlights the complexity of bacterial resistance in wild animals, which act as reservoirs and vectors of resistant bacterial pathogens, and the possible interspecies transmission among humans, domestic animals, the environment, and wildlife [17]. Therefore, new health problems in wildlife populations have emerged, and novel reservoirs of zoonotic diseases have formed.

The aim of this study was to obtain a detailed understanding of the possibility that migratory and non-migratory wild birds can harbour and spread colistin-resistant bacteria, which could pose a potential risk to human and animal health by transmission of antimicrobial-resistant strains to waterways and environmental sources through faecal deposits.

## Materials and methods

### Sample preparation

**Fig. 1:**
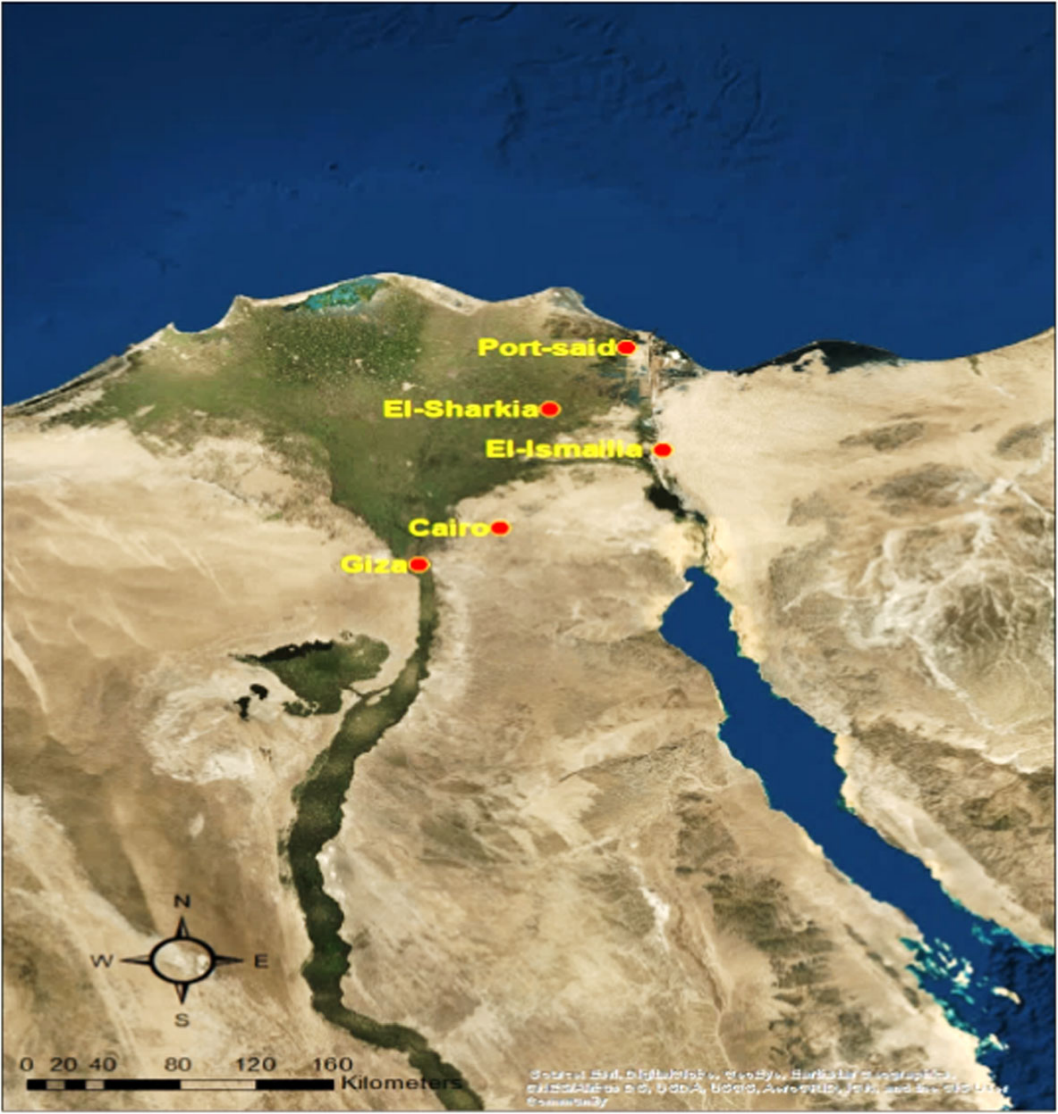
Location of sampling points in Egypt

A total of 140 faecal samples were collected from 80 resident wild birds (20 hooded crows, 20 cattle egrets, 20 rock pigeons and 20 laughing dove) and 60 migratory waterfowls (20 shoveler ducks, 20 Cotte ducks and 20 green-winged teal ducks) were collected from Giza, Cairo, El-Sharkia, El-Ismailia and Port-said governorates in Egypt (Fig. 1) during the period from August 2017 to January 2018. Modified traps were used to capture wild birds and migratory waterfowls during winter migration. After trapping, faecal swabs were taken, and the birds were released. The swabs were then placed in 2 ml of sterile saline (0.9% NaCl) and stored in an ice box until being transported to the laboratory.

Twenty Surface water samples from 20 different sources (1 from each) were collected in sterile glass bottles in the area in which wild-bird trapping was conducted. Upon arrival, all water samples were filtered using a membrane filtration method, which was carried out according to American Public Health Association (APHA) [19]. In this procedure, 100-ml water samples were drawn through a vacuum filtration apparatus containing a sterile filter membrane with a pore size of 0.45 μm (Sartorius, France), which retained bacteria, and then, the filters were vortexed in peptone broth to recover the bacteria. After removing the filters, the bacteria were cultivated at 37 °C for 24 h.

Fifty human stool specimens were gathered from apparently healthy farmers residing near the surface water sources and farm buildings in the area in which wild-bird trapping was conducted. Samples were taken with sterile swabs, and the swabs were transferred to tubes containing trypticase soy broth and incubated at 37 °C for 24 h.

### Bacterial isolation and identification

A loopful of all the previously inoculated broths was cultured on eosin methylene blue (EMB) agar (Oxoid, Oxoid Ltd., London) for isolation of *Enterobacteriaceae* other than salmonellae*.* Pseudomonas agar base with CN supplement (Oxoid) was used for the selective isolation of *P. aeruginosa*. All the inoculated plates were incubated aerobically at 37 °C for 24 h. Suspected colonies were picked and examined for morphological and culture characteristics according to Quinn et al. [20].

The API 20E and API 20NE kits (BioMerieux, France) were used according to the manufacturer’s instructions to detect the biochemical profiles of the isolated *Enterobacteriaceae* and *P. aeruginosa*, respectively.

### Molecular identification *mcr-1* and *mcr-2*

All isolates obtained from the examined samples were subjected to genotyping using multiplex polymerase chain reaction (PCR) according to Cavaco et al. [21].

The template DNA used consisted of boiled lysates prepared from the isolates. In brief, a loopful of culture was suspended in 100 μl of sterile TE buffer, boiled for 10 min at 100 °C, and centrifuged for 5 min at 6000×g. The extracted DNA was stored at − 20 °C until use.

PCR was performed on the extracted DNA, wherein two genes were targeted as described in Table (1). PCR amplification was performed using 2 μl of the DNA template, 25 μl of 2× DreamTaq DNA PCR Master Mix (Thermo Scientific, Waltham, USA), and 2 μl of each primer at a concentration of 2 μM, and nuclease-free water was added up to 50 μl.

Additionally, PCR reaction was done using *E. coli* carrying *mcr-1* (ID: 2012-60-1176-27) and *E. coli* containing *mcr-2* (ID: KP37) as a positive control, while water sample was used as a negative control.

The thermal profile of the reaction was as follows: initial denaturation at 94 °C for 15 min, 25 cycles of denaturation at 94 °C for 30 s, annealing at 58 °C for 90 s, and extension at 72 °C for 60 s, followed by a final extension step at 72 °C for 10 min.

Then, gel electrophoresis of the amplified PCR product was performed on a 1.5% agarose gel, which was visualized under ultraviolet light.

### Sequence analysis

The amplified fragments of *mcr*-1 gene from *E. coli* carrying *mcr-*1 isolates that randomly selected from migratory bird, human and water in the same locality were purified using the QIAquick Gel Extraction Kit (QIAGEN, Germany) according to the manufacturer’s instructions and sequenced at Promega Lab Technology (Germany) using the forward and reverse primers of the *mcr-1* gene listed in Table 1.

**Table 1:** List of primer pairs used for the *mcr-1* and *mcr-*2 genes in this study

Target gene	Primary sequences	Amplicon size	Reference
*mcr*-1 (35–343)	CLR F 5′-CGGTCAGTCCGTTTGTTC-3′ CLR R 5′-CTTGGTCGGTCTGTAGGG-3	309 bp	[22]
*mcr*-2 (494–1060)	MCR2 IF 5′- TGTTGCTTGTGCCGATTGGA-3′ MCR2 IR 5′-AGATGGTATTGTTGGTTGCTG-3’	567 bp	[23]

The gene sequences have been deposited in the National Center for Biotechnology Information (NCBI) GenBank database under the accession numbers MK 530689, MK 620991, MK 620992 for the water, human stool, and migratory bird-derived sequences, respectively.

The genes sequenced in this study were compared with the sequences available in the public domain using the NCBI BLAST server. Publicly available gene sequences were downloaded from NCBI GenBank and aligned using CLUSTALW in BioEdit version 7.0.1.4. Phylogenetic analysis was performed with MEGA version 7 using the neighbour-joining approach. The bootstrap consensus tree was estimated from 1000 replicates.

### Ethics statement

Protocols for the collection of samples were conducted in accordance with applicable legislation of the Institutional Animal Care and Use Committee, of the Faculty of Veterinary Medicine, Cairo University, Egypt.

Oral consent was obtained from each abattoir worker participated in the study upon information on the use of hand swab samples.

## Result

In total, 140 wild birds were sampled. Bacteriological examination revealed that all the wild birds sampled carried members of the family *Enterobacteriaceae*, including *E. coli*, *K. pneumoniae*, and *Klebsiella oxytoca*. *P. aeruginosa*, as a *non-Enterobacteriaceae* species, was also isolated (Table 2). The occurrence of isolated bacterial species varied among host types (Table 3). Moreover, *E. coli*, *K. pneumoniae*, *K. oxytoca* and *P. aeruginosa* were also isolated from water and stool samples.

**Table 2:** Prevalence of the bacteria isolated from wild birds, water and humans

Sample		Total number	Isolated bacteria			
			*E. coli*	*K. pneumoniae*	*K. oxytoca*	*P. aeruginosa*
Resident birds	Hooded crow	20	12 (60%)	4 (20%)	1 (5%)	1 (5%)
	Cattle egret	20	9 (45%)	1 (5%)	0 (0%)	0 (0%)
	Rock pigeon	20	10 (50%)	3 (15%)	1 (25%)	3 (15%)
	Laughing dove	20	11 (55%)	11 (55%)	2 (10%)	4 (20%)
	Total	80	33 (41.2%)	22 (27.5%)	4 (5%)	8 (10%)
Migratory birds	Shoveler duck.	20	9 (45%)	4 (20%)	1 (5%)	6 (30%)
	Cotte duck	20	12 (60%)	3 (15%)	2 (10%)	2 (10%)
	Green-winged teal duck	20	8 (40%)	2 (10%)	3 (15%)	3 (15%)
	Total	60	29 (48.3%)	9 (15%)	6 (10%)	11 (18.3%)
Water		20	7 (35%)	7 (35%)	1 (5%)	3 (15%)
Humans		50	10 (20%)	15 (30%)	2 (4%)	4 (8%)

**Table 3:** Frequencies of colistin resistance genes among different bacterial isolates from different isolation sources

Sample		Total isolates	Colistin resistance genes		
			*mcr*-1	*mcr*-2	Both
Resident birds	*E. coli*	33	3 (9.1%)	0	0
	*K. pneumoniae*	22	1 (4.5%)	0	0
	*K. oxytoca*	4	0	0	0
	*P. aeruginosa*	8	3 (37.5%)	1 (12.5%)	0
	Total	67	7 (10.4%)	1 (1.4%)	0
Migratory birds	*E. coli*	29	6 (20.6%)	1 (3.4%)	1 (3.4%)
	*K. pneumoniae*	9	2 (22.2%)	1 (11.1%)	1 (11.1%)
	*K. oxytoca*	6	0	0	0
	*P. aeruginosa*	11	3 (27.2%)	0	0
	Total	55	11 (20%)	2 (3.6%)	2 (3.6%)
Water	*E. coli*	7	2 (28.5%)	1 (14.2%)	0
	*K. pneumoniae*	7	1 (14.2%)	1 (14.2%)	0
	*K. oxytoca*	1	0	0	0
	*P. aeruginosa*	3	0	0	0
	Total	18	3 (16.6%)	2 (11.1%)	0
Humans	*E. coli*	10	1 (10%)	1 (10%)	0
	*K. pneumoniae*	15	1 (6.6%)	2 (13.3%)	1 (6.6%)
	*K. oxytoca*	2	0	0	0
	*P. aeruginosa*	4	0	0	0
	Total	31	2 (6.4%)	3 (9.6%)	1 (3.2%)

The *mcr* genes were detected in various *E. coli, K. pneumoniae* and *P. aeruginosa* isolates recovered from resident birds and migratory birds, while in this study, the *mcr-1* and *mcr-2* genes were detected in only *E. coli* and *K. pneumoniae* isolates from water sources and human stool samples. In this study, *E. coli* was the most prevalent gram-negative bacterial species harbouring the *mcr* genes, whereas a low prevalence was observed in *K. pneumoniae* and no *mcr* genes isolated from *K. oxytoca*.

**Fig. 2:**
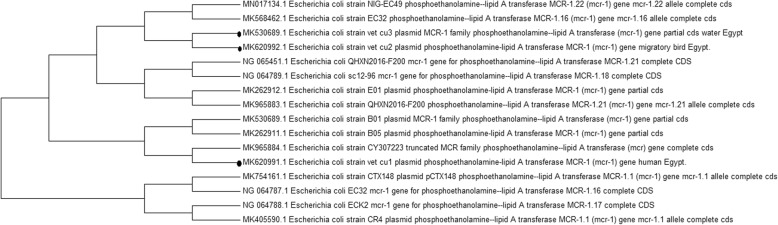
Neighbour joining tree showing the relationship between the nucleotide sequences of the partial coding regions of *mcr-1* gene. The studied sequences were remark by bullets. The Evolutionary analysis was performed with MEGA version 7

Comparing the sequences of the *mcr-1* gene revealed 100% homology between the selected isolates from the wild bird and water isolates, and lack of homology with the human isolate as shown in Fig. 2.

## Discussion

The emergence of new infectious diseases in wildlife [24, 25] and the potential threat of these diseases as zoonoses has increased the general interest in birds as vectors of pathogens and their role in disease epidemiology. The lifestyles of migratory birds allow them to carry and disseminate pathogenic and resistant microorganisms across country borders.

In this study, bacteriological examination of resident and migratory birds revealed the presence of *E. coli, K. pneumoniae, K. oxytoca* and *P. aeruginosa.* In addition, these bacteria were also recovered from water and human samples.

Special consideration for crows was consistent with Lee et al. [26] who showed that the house crow usually lives close to humans. This bird occupies different ecological niches, including household areas, city dumps, hospital dumps, and water sources such as lakes, ponds, and rivers. Many bird species have been found to carry antibiotic-resistant bacteria. Resistant *E. coli* have been isolated from ducks, geese [17, 27], cormorants [28, 29], birds of prey [30], gulls [31], doves [32], and passerines [33].

When tracking the source of the *mcr*-1 gene in previous studies, it was closely related to human activities and movement, while environmental dissemination of this gene was rarely considered and much less the role that migratory birds might play in the process [34, 35].

The first known occurrence of the *mcr-*1 gene was in *E. coli* carried by a wild migratory bird, namely, the European herring gull (*Larus argentatus*) [36], and the first detection of *mcr*-1 was in colistin-resistant extended-spectrum β-lactamase-producing *E. coli* (ESBL-*E. coli*) isolated from the wild transboundary migratory waterfowl species *Fulica atra* from Pakistan, Asia [37].

The current study showed that *mcr* genes were detected in *E. coli, K. pneumoniae* and *P. aeruginosa* from resident birds and migratory birds (Table 2). The prevalence of *mcr-*1 in resident birds, migratory birds were 10.4, 20% while the prevalence of *mcr*-2 were 1.4, 3.6%, respectively. This occurrence of *mcr* genes in bacterial isolates of resident and migratory birds was surprising. This is because these findings were much higher than that obtained by Ruzauskas et al.*,* [36] who found that the percentage of *mcr-*1 gene were really low (0.85%) in 117 *E. coli* isolates recovered from 160 European herring gulls.

*E. coli* remains the most prevalent gram-negative bacterium harbouring the *mcr* gene, and these results are consistent with Skov and Monnet [38]. whereas low prevalence was observed in *K. pneumoniae*; however, the latter pathogen is a leading cause of nosocomial infections globally.

The emergence of resistant bacteria in wildlife may be related to the passage of antibiotic resistance genes to the environment [39]. The possibility of water contamination by bird faeces should be considered an important risk factor for the transmission of resistant bacteria [40, 41].

In this study, the *mcr-1* and *mcr-2* genes isolated from water sources were 16.6 and 11.1, respectively and these genes were detected only from in *E. coli and K. pneumoniae* isolates. This result agreed with Wu et al. [41] who detected the *mcr-1* gene in three colistin-resistant *E. coli* isolates, among which two were isolated from river water and one from egret faeces.

The occurrence of *mcr* genes in bacteria isolated from farmers residing near the area from which surface water and wild bird sampling conducted was confirmed in our study. The persistence of *mcr*-1 in the human GI tract microflora can cause further contamination of water systems through disposal of waste water containing human faeces [42].

There is no doubt that human intervention plays a substantial role in the development of AMR, as there is a linear correlation between the use of antibiotics and the development of resistance [43]. The zoonotic potential of *mcr*-1-carrying bacteria has been indicated by many studies [42, 44, 45]. In addition, the dissemination of *mcr*-carrying bacteria from wild birds and other livestock animals to humans could occur via the food chain or direct contact with animals as well as through contamination of fresh and seawater systems [10, 46].

To determine the correlation between wild birds, water sources and humans, phylogenetic analysis using *mcr-1* genes isolated from these sources was performed. The sequences of the *mcr*-1 genes revealed 100% homology between the sequences from water source and wild-bird isolates, and no homology with human isolates as shown in Fig. 2. This finding indicated the dissemination of this gene between both sources. Moreover, this finding confirmed the role of wild birds in antibiotic resistance as they consider a potential spreader of antibiotic resistance through the ability to migrate long distances in short periods of time and its effect on the environment contamination.

Most of the existing research results have revealed that the dissemination of environmental AMR is closely related to anthropogenic factors. Bacteria seldom develop AMR in regions free from antibiotic pressure and human activities [47]. Moreover, scientists have speculated that migratory animals, especially migratory birds, may carry resistant bacteria or genes and transport them to regions far from anthropogenic influences [48].

The clear limitation of this study was only three isolates were sequenced. Further studies on colistin-resistance co-exists with other resistance patterns (such as carbapenem-resistance) is planned.

## Conclusion

This study is the first to detect of *mcr*-1-positive bacteria in wild birds in Egypt, indicating the possibility of environmental dissemination of this gene through bird activity. In addition to their impact on the environment, the levels of resistance in wild birds tend to correlate with the degree of contact with human activities as they easily accumulate human and environmental bacteria. Zoonotic infections caused by bacteria carrying *mcr-1* highlight the urgent need to limit the use of colistin in agriculture and veterinary practices.

## Data Availability

All data generated or analysed during this study are included in this published article.
